# Supplementary material for: An attribute-enhanced relationship-aware neighborhood matching model with dual attention
Source: PLoS One. 2025 Jun 2;20(6):e0324290. doi: 10.1371/journal.pone.0324290 (PMC12129319; doi:10.1371/journal.pone.0324290)
Supplement: Attachment — Submitted filename: response letter.docx [file pone.0324290.s001.docx]

**Response to Review Comments**

**Manuscript ID:** PONE-D-24-50383

**Title:** An Attribute-Enhanced Relationship-Aware Neighborhood Matching Model with Dual Attention

**Authors:** Junlin Gu, Weiwei Liu, Xiong Yang

We would like to thank the editor for handling the review process of our manuscript. We are very grateful to the reviewers for their thorough review and constructive comments. We have carefully addressed all the review comments and improved the quality of this manuscript accordingly. Please find below our response to the review comments one-by-one.

**Response to Reviewer 1’s Comments**

**Comment 1:** *Suggest adding some references published in the past two years.*

**Response: Done.** Thanks for the reviewer’s constructive comment. Following this comment, we have supplemented three references published in the past two years.

**Supplemented references:**

[4] Zhu L, Li N, Bai L. Embedding-based entity alignment between multi-source temporal knowledge graphs. Engineering Applications of Artificial Intelligence.2024:133:108451.

[5] Masmoudi M, Ben Abdallah Ben Lamine S, Karray MH, Archimede B, Baazaoui Zghal H. Semantic data integration and querying: a survey and challenges. ACM Computing Surveys. 2024; 56(8): 1-35.

[7] Zhu B, Wang R, Wang J, Shao F, Wang K. A survey: knowledge graph entity alignment research based on graph embedding. Artificial Intelligence Review. 2024; 57(9): 229.

**Comment 2:** *Suggest adding some advanced baselines for comparison.*

**Response: Done.** Thanks for the reviewer’s constructive comment. Following this comment, we have supplemented two advanced baselines that are more relevant to our work in Section 5.3.1. Specially, Dual-AMN [37] employs the KG encoder dual-attention matching network, which not only intelligently models intra- and cross-graph relationships but also greatly reduces computational complexity. MSENEA [38] makes comprehensive use of multi-modal knowledge by using intermodal effects to align entities between multi-modal KGs.

**Supplemented references:**

[37] Mao X, Wang W, Wu Y, Lan M. Boosting the speed of entity alignment 10xDual attention matching network with normalized hard sample mining. In: Proceedings of the web conference 2021; 2021. p. 821-832.

[38] Chen L, Li Z, Xu T, Wu H, Wang Z, Yuan NJ, et al. Multi-modal Siamese network for entity alignment. In: Proceedings of the 28th ACM SIGKDD conference on knowledge discovery and data mining; 2022. p. 118-126.

**Comment 3:** *The format of the table in the manuscript seems to be incorrect, it is recommended to check.*

**Response: Explained.** Thanks for the reviewer’s suggestion. Although the format of the table in the manuscript seems to be incorrect, we wrote the manuscript strictly in accordance with the official PLOS ONE latex template, which has the tables in that format. Therefore, we continue to write the tables according to the official template.

**Comment 4:** *There are still some typos in the manuscript.*

**Response: Corrected.** Following this detailed comment, we have carefully checked the manuscript and improved its quality. For example, in the entire manuscript, “relation-aware” has been standardized as “relationship-aware”. In the abstract, “the” has been supplemented before four modules. In the 1^st^ paragraph of Section 1, “the real-world” has been revised to “real-world” and “even” has been revised to “are”. In Section 2.1, “neighbor-hood” has been revised to “neighborhood”. In the last paragraph of Section 3, “$e_1$” has been revised to “$e_2$”. In the 1^st^ paragraph of Section 4, “a” has been supplemented before “dual”. In Table 1, “tripes” has been revised to “triples”.

**Response to Reviewer 2’s Comments**

**Comment 1:** *Expand Evaluation: Include experiments on more diverse and domain-specific datasets.*

**Response: Done.** Thanks for the reviewer’s constructive comment. Following this comment, we have supplemented a new dataset SRPRS in Section 5.1. Specially, since the DBP15K dataset is too dense and the degree distribution is very different from the real-world data, we also chose two cross-linguistic subsets from the relatively sparse SRPRS dataset [33], and comprehensive dataset details are presented in Table 2.

**Supplemented references:**

[33] Guo L, Sun Z, Hu W. Learning to Exploit Long-term Relational Dependencies in Knowledge Graphs. In: Chaudhuri K, Salakhutdinov R, editors. Proceedings of the 36th International Conference on Machine Learning. vol. 97; 2019. p. 2505-2514.

**Comment 2:** *Add Baselines: Compare the model against newer methods, such as transformer-based or hyperbolic embedding approaches.*

**Response: Done.** Thanks for the reviewer’s constructive comment. Following this comment, we have supplemented two advanced baselines that are more relevant to our work in Section 5.3.1. Specially, Dual-AMN [37] employs the KG encoder dual-attention matching network, which not only intelligently models intra- and cross-graph relationships, but also greatly reduces computational complexity. MSENEA [38] makes comprehensive use of multi-modal knowledge by using intermodal effects to align entities between multi-modal KGs.

**Supplemented references:**

[37] Mao X, Wang W, Wu Y, Lan M. Boosting the speed of entity alignment 10xDual attention matching network with normalized hard sample mining. In: Proceedings of the web conference 2021; 2021. p. 821-832.

[38] Chen L, Li Z, Xu T, Wu H, Wang Z, Yuan NJ, et al. Multi-modal Siamese network for entity alignment. In: Proceedings of the 28th ACM SIGKDD conference on knowledge discovery and data mining; 2022. p. 118-126.

**Comment 3:** *Reproducibility: Provide a link to the implementation or specify more details about the experimental environment.*

**Response: Explained.** Thank the reviewer for their attention to code reproducibility. We attach great importance to the reproducibility of the experimental results and describe the algorithmic process, parameter settings, and experimental details in the paper. As the project involves some commercial cooperation and confidentiality requirements, we do not plan to disclose the source code yet. However, we are willing to provide the necessary technical support and detailed descriptions within reasonable limits to ensure that our peers can understand and reproduce the results of our work. We will also continue to pay attention to the feedback from the community, and if conditions permit, we will consider releasing part of the code to promote academic exchanges.

**Comment 4:** *Highlight Practical Use Cases: Discuss* *potential real-world applications or case studies where this model could be impactful.*

**Response: Done.** Following this suggestion, we have supplemented Section 6 Potential applications, which discusses potential real-world applications. Specially, the proposed ARNM-DAE2A model, with its ability to effectively align entities across different KGs, has potential applications in several fields, including intelligent search and question answering, enterprise knowledge management and data integration, recommendation systems, and multilingual knowledge graph integration.

**Response to Reviewer 3’s Comments**

**Comment 1:** *The abstract could be improved by explicitly discussing the motivation for using dual attention mechanisms and the significance of integrating attribute information.*

**Response: Done.** Thanks for the reviewer’s constructive comment. Following this comment, we have supplemented the motivation for using dual attention mechanisms and the significance of integrating attribute information. Specifically, traditional graph-based methods often lose information due to insufficient use of attributes and imperfect relationship modeling, which makes it difficult to capture the deep semantic relationship between entities fully. To improve the effect of entity alignment, we propose a new model named ARNM-DAE2A, which strengthens the information aggregation capability of GCN by introducing a dual-attention mechanism to ensure a more balanced and comprehensive structural representation.

**Comment 2:** *Despite emphasizing the role of attributes in the introduction, the method simply uses attributes as GNN inputs rather than actively leveraging them in interaction with the structural embeddings.*

**Response: Revised.** Thanks for the reviewer’s suggestion. We have supplemented the design ideas of the joint alignment module in Section 4.1 by highlighting the ways in which attribute and structural information can be interacted. Specially, After obtaining the entity embeddings based on the relationship structure and attribute structure, we not only compute the similarity between the two separately, but also design a cross-interaction fusion layer, which enables the attribute embeddings and structural embeddings to actively complement each other and learn collaboratively through the introduction of a cross-attention mechanism, so that we can obtain a more comprehensive and robust entity representation. Moreover, we also recognize that the current fusion strategy is relatively simple and have supplemented future work in Section 7. Specially, in future work, we plan to explore more proactive fusion mechanisms, such as cross-modal attention or dynamic feature selection based methods, to enable attribute embedding to adaptively adjust the weights between different entity pairs and improve the robustness of alignment.

**Comment 3:** *Some hyperparameters (e.g., seed alignment ratio and hyperparameters in Equation 17) are arbitrarily set without detailed discussion or justification.*

**Response: Explained.** Thanks for the reviewer’s constructive comment. Except for the hyperparameters set in the references, all hyperparameter settings are optimized by experimental tests. We have supplemented the references in Section 5.2.3.

**Comment 4:** *The experiments are conducted only on the DBP15K dataset. The model could be potentially evaluated on other, more diverse datasets (e.g., Wikidata, YAGO) as well.*

**Response: Done.** Thanks for the reviewer’s constructive comment. Following this comment, we have supplemented a new dataset SRPRS in Section 5.1. Specially, since the DBP15K dataset is too dense and the degree distribution is very different from the real-world data, we also chose two cross-linguistic subsets from the relatively sparse SRPRS dataset [33], and comprehensive datasets detail are presented in Table 2.

**Supplemented references:**

[33] Guo L, Sun Z, Hu W. Learning to Exploit Long-term Relational Dependencies in Knowledge Graphs. In: Chaudhuri K, Salakhutdinov R, editors. Proceedings of the 36th International Conference on Machine Learning. vol. 97; 2019. p. 2505-2514.

**Comment 5:** *In the ablation experiments, some configurations (e.g., MM+AE) outperform the final model and may require some discussions or explanations.*

**Response: Done.** Thanks for the reviewer’s constructive comment. Following this comment, we have supplemented discussions in the last paragraph of Section 5.3.2. Specially, MM+AE yields impressive results, occasionally even surpassing the overall model’s performance. This is because that attribute feature can in some cases provide very different discriminative information from the graph structure, allowing the matching module to be more targeted in correcting initial misalignments. However, Hits@10, Hits@50, or MRR do not necessarily outperform the full model at the same time, suggesting that relying on attribute features alone may also be a bottleneck in a wider range of entity retrievals. Once the attributes are inadequate or noisy, the advantage of the AE module is diminished and cannot replace the value of structural embedding for global information capture.

**Comment 6:** *The model is quite complex. Discussions or empirical analysis on its scalability are required.*

**Response: Done.** Following this suggestion, we have performed a detailed derivation of the computational complexity of the model in Section 4.5, illustrating how the overall computational complexity varies with data size, number of network layers, and embedding dimensionality based on each module. Although the model structure is more complex, in theory, its complexity is the same as that of the existing GCN-based methods. Moreover, to further verify the model’s scalability, we have conducted experiments on the relatively sparse dataset SRPRS in Section 5.1. The experimental results show that despite the challenges posed by the sparse data, our model still maintains a good performance, which proves that the model has good scalability and adaptability. In addition, we have recorded the model’s training time and compared it with other methods in the last paragraph of Section 5.3.1. The results show that while our method’s training time is slightly longer, this increment is exchanged for higher alignment accuracy and robustness, thus validating the model’s acceptability for real-world applications.

**Comment 7:** *The baselines used in the experiments are relatively old. The most recent baseline is from 2020. There are more recent methods that can be compared as baselines.*

**Response: Done.** Thanks for the reviewer’s constructive comment. Following this comment, we have supplemented two advanced baselines that are more relevant to our work in Section 5.3.1. Specially, Dual-AMN [37] employs the KG encoder dual-attention matching network, which not only intelligently models intra- and cross-graph relationships but also greatly reduces computational complexity. MSENEA [38] makes comprehensive use of multi-modal knowledge by using intermodal effects to align entities between multi-modal KGs.

**Supplemented references:**

[37] Mao X, Wang W, Wu Y, Lan M. Boosting the speed of entity alignment 10xDual attention matching network with normalized hard sample mining. In: Proceedings of the web conference 2021; 2021. p. 821-832.

[38] Chen L, Li Z, Xu T, Wu H, Wang Z, Yuan NJ, et al. Multi-modal Siamese network for entity alignment. In: Proceedings of the 28th ACM SIGKDD conference on knowledge discovery and data mining; 2022. p. 118-126.

**Comment 8:** *A minor typo in Table 1 "tripes."*

**Response: Corrected.** Following this detailed comment, we have carefully checked the manuscript and improved its quality. For example, in the entire manuscript, “relation-aware” has been standardized as “relationship-aware”. In the abstract, “the” has been supplemented before four modules. In the 1^st^ paragraph of Section 1, “the real-world” has been revised to “real-world” and “even” has been revised to “are”. In Section 2.1, “neighbor-hood” has been revised to “neighborhood”. In the last paragraph of Section 3, “$e_1$” has been revised to “$e_2$”. In the 1^st^ paragraph of Section 4, “a” has been supplemented before “dual”. In Table 1, “tripes” has been revised to “triples”.

**Comment 9:** *The source code is not shared.*

**Response: Explained.** Thank the reviewer for their attention to code reproducibility. We attach great importance to the reproducibility of the experimental results and describe the algorithmic process, parameter settings, and experimental details in the paper. As the project involves some commercial cooperation and confidentiality requirements, we do not plan to disclose the source code yet. However, we are willing to provide the necessary technical support and detailed descriptions within reasonable limits to ensure that our peers can understand and reproduce the results of our work. We will also continue to pay attention to the feedback from the community, and if conditions permit, we will consider releasing part of the code to promote academic exchanges.

**Response to Reviewer 4’s Comments**

**Comment 1:** *While the manuscript is well-written in standard English, some sections could benefit from clearer and more concise explanations. For instance, the description of the dual attention mechanism, while technically sound, might be challenging for readers unfamiliar with the concept. Including illustrative examples or visual aids could greatly enhance comprehension and accessibility.*

**Response: Done.** Thanks for the reviewer’s constructive comment. Following this comment, we have supplemented an illustrative example in the Section 5.3.5 Case Study. Specially, we take Fig. 1 as an example of a case study of the workflow of the dual attention mechanism. While the traditional one-way attention mechanism only passes information from entities to their neighbors, our dual attention mechanism calculates two sets of attention weights simultaneously. On the one hand, in the Chinese KG, by calculating the source attention from a Chinese-side entity to its neighboring nodes, the importance of each neighbor to the representation of that entity can be quantified; on the other hand, by calculating the target attention from the neighboring nodes back to that Chinese-side entity, the feedback contribution of the neighbors to the representation of the entity is reflected. Similarly, in the English KG, the exact bidirectional computation is performed for the corresponding English-side entity and its neighbor nodes. Subsequently, these two attention weights are fused to form a more comprehensive and robust entity representation. Based on this fused representation, the alignment process shown in Fig. 1 can accurately capture the semantic consistency between the two sides of the KG in terms of languages, population, capital, and central bank, significantly improving the accuracy of entity alignment and the model's noise immunity.

**Comment 2:** *Additionally, the practical implications and potential real-world applications of the proposed method deserve more attention. Expanding on how this approach could be applied to larger or more heterogeneous knowledge graphs, or how it handles noisy or incomplete data, would highlight its broader relevance and strengthen its appeal to a wider audience.*

**Response: Done.** Thanks for the reviewer’s constructive comment. Following this comment, we have supplemented a new dataset SRPRS in Section 5.1. Specially, since the DBP15K dataset is too dense and the degree distribution is very different from the real-world data, we also chose two cross-linguistic subsets from the relatively sparse SRPRS dataset [33], and comprehensive dataset details are presented in Table 2. In addition, we have supplemented the robustness with textual noise in the 2^nd^ paragraph of Section 5.3.4. Specially, referring to [39], we test the robustness of the model by artificially introducing different degrees of textual noise, where textual noise includes entity errors, missing words, and additional characters. As shown in Fig. 8, the alignment accuracy of each dataset tends to decrease as the proportion of noise increases, but the magnitude of the decrease is significantly different. In the DBP15K subsets, Hits@1 decreases more gently, which indicates that the model can rely on sufficient structural and attribute information to mitigate the negative impact of noise in the case of richer information. In the SRPRS subsets, the decrease in Hits@1 is more pronounced due to the sparser graphs and less redundant information. This suggests that textual perturbations interfere more with entity alignment in noisy scenarios with sparse data. Overall, the experimental results illustrate that the proposed ARNM-DAE2A is robust under low to moderate noise levels, but there is still room for improvement under high noise and sparse data conditions, and further optimization of the noise processing and information fusion strategies is needed. Moreover, we have supplemented a new section, Section 6 Potential applications, where we discuss real-world applications for our model to enhance its appeal.

**Supplemented references:**

[33] Guo L, Sun Z, Hu W. Learning to Exploit Long-term Relational Dependencies in Knowledge Graphs. In: Chaudhuri K, Salakhutdinov R, editors. Proceedings of the 36th International Conference on Machine Learning. vol. 97; 2019. p. 2505-2514.

[39] Yoon S, Ko S, Kim T, Kang S, Yeo J, Lee D. Unsupervised Robust Cross-Lingual Entity Alignment via Neighbor Triple Matching with Entity and Relation Texts; 2025. Available from: https://arxiv.org/abs/2407.15588.

**Response to Reviewer 5’s Comments**

**Comment 1:** *First and foremost, I recommend the authors publish their code in a git repository such as GitHub. This would substantially improve usability of their model for other researchers.*

**Response: Explained.** Thank the reviewer for their attention to code reproducibility. We attach great importance to the reproducibility of the experimental results and describe the algorithmic process, parameter settings, and experimental details in the paper. As the project involves some commercial cooperation and confidentiality requirements, we do not plan to disclose the source code yet. However, we are willing to provide the necessary technical support and detailed descriptions within reasonable limits to ensure that our peers can understand and reproduce the results of our work. We will also continue to pay attention to the feedback from the community, and if conditions permit, we will consider releasing part of the code to promote academic exchanges.

**Comment 2:** *The mathematical formulation of the model is rigorously documented, but the relation between the notation and Figure 1 seems incompletely explored. A short explanation which relates the figure back to the notation could enhance readability for readers unfamiliar with the problem domain. Similarly, it may help to clarify what the characters on the left-hand-side of Figure 1 mean for readers who do not know the language. As someone who does not speak or read what I presume to be Mandarin, I can only assume that the entities on the left hand side are direct translations of those same entities on the right hand side.*

**Response: Done.** Following this suggestion, we have supplemented description of Fig. 1 in the last paragraph of Section 3. Specially, Fig. 1 shows an example of cross-language entity alignment, where the left nodes are from the Chinese KG (labels are presented in Chinese), and the right nodes are from the English KG (labels are presented in English). Each node on the left is a direct translation of its corresponding node on the right. In the mathematical description, the symbol $E$ denotes the set of all entities in the two KGs, and this correspondence forms the basis of the interaction between structure and attribute information in the alignment process of this model.

**Comment 3:** *In section 5.1, I believe that it could be helpful to note that the train/test split used was an exact match to the split used in prior studies. This is cited by Wu et al. in their paper on RDGCN as originating with Sun, 2018.*

**Response: Explained.** We carefully checked the experimental setup of RDGCN and found that the training/test set division ratio we used is identical to the one used in the RDGCN. Therefore, our experimental setup is consistent with previous work to ensure comparable results. To illustrate this point, we have supplemented the description in Section 5.1. Specially, for comparison with previous works, we used the same training/testing split with previous works, 30% for training and 70% for testing.

**Comment 4:** *On page 5, there may be a typo which states "e_1 denotes another instance"; based on context, I believe that this is intended to be e_2 instead.*

**Response: Corrected.** Following this detailed comment, we have carefully checked the manuscript and improved its quality. For example, in the entire manuscript, “relation-aware” has been standardized as “relationship-aware”. In the abstract, “the” has been supplemented before four modules. In the 1^st^ paragraph of Section 1, “the real-world” has been revised to “real-world” and “even” has been revised to “are”. In Section 2.1, “neighbor-hood” has been revised to “neighborhood”. In the last paragraph of Section 3, “$e_1$” has been revised to “$e_2$”. In the 1^st^ paragraph of Section 4, “a” has been supplemented before “dual”. In Table 1, “tripes” has been revised to “triples”.

**Response to Reviewer 6’s Comments**

**Comment 1:** *The manuscript lacks a rigorous theoretical discussion explaining why the dual attention mechanism and attribute-enhanced embeddings contribute to improved performance. While the empirical results demonstrate effectiveness, a deeper theoretical justification would significantly strengthen the contribution.*

**Response: Done.** Thanks for the reviewer’s suggestion. Following this suggestion, we have supplemented and analyzed why the dual attention mechanism and attribute-enhanced embeddings contribute to improved performance in the 1^st^ paragraph of Section 4.1 and Section 4.2, respectively. Specially, in the traditional GCN, information transfer is primarily based on unidirectional neighborhood aggregation, which is susceptible to neighborhood noise and information asymmetry problems, resulting in less robust node representations. If information can be aggregated from both source and target nodes at the same time, i.e., bi-directional information interaction is realized, it helps improve the symmetry and stability of information transfer. Therefore, this module introduces the dual-attention mechanism to theoretically achieving noise suppression and key information reinforcement by adaptively assigning neighborhood weights, thereby improving the overall entity representation quality. Moreover, in real KGs, it is often difficult to rely on structural information alone to distinguish entities with similar connection patterns but subtle semantic differences. The attribute data of entities, such as names, descriptions, and other semantic labels, can provide complementary information that enhances the discriminative power of entity representations. Based on this, this module uses an independent GCN to model the entity attributes and vectorize the extracted attribute features. Attribute information has a high degree of discrimination, which can distance structurally similar but semantically different entities in the embedding space, making it easier for the model to capture pairs of entities that are truly semantically related when aligned. With attribute-enhanced embedding, we hope to use this additional information to compensate for the limitations of single-structure embedding and further improve the overall discriminative power and alignment accuracy of the model.

**Comment 2:** *The proposed model integrates multiple components, including GCN-based entity structure learning, attention mechanisms, and neighborhood-aware matching. However, the manuscript does not provide a detailed analysis of the computational cost associated with each component.*

**Response: Done.** Following this suggestion, we have supplemented complexity analysis in Section 4.5. Specially, GCN is responsible for learning the structured representation of entities, and the main computational effort is from neighborhood feature aggregation and feature transformation. Assuming that a KG contains $N$ entities and $E$ edges with embedding dimension $d$ and GCN adopts $L$ layers, the complexity of neighborhood feature aggregation is $O(Ed)$, the complexity of feature transformation is $O(Nd^{2})$, and the overall computational complexity is $O(L(Ed+Nd^{2}))$. The dual attention mechanism is used to optimize the GCN propagation process by introducing additional attention weight calculation and weighted neighbor aggregation. Assuming that the average number of neighbors of each entity is $k$, the complexity of attention computation is $O(Nkd)$ and the complexity of weighted aggregation is also $O(Nkd)$, and the overall complexity is $O(LNkd)$. The joint alignment module calculates the entity similarity by fusing structural and attribute information, and the computational complexity is determined by the embedding fusion and similarity calculation. Here, the computational complexity of the fusion process is $O(Nd)$, the complexity of similarity computation is $O(Md)$ based on the number of entity pairs $M$, and the overall complexity is $O(Nd+Md)$. The relationship-aware neighborhood matching module further adjusts the entity alignment results, and its computational complexity is primarily determined by the neighborhood matching computation. Here, the complexity of the neighborhood matching operation for each entity is $O(Nkd)$. In summary, the overall computational complexity of ARNM-DAE2A is $O\left( L\left( Ed+Nd^{2} \right) \right)+O\left( LNkd \right)+O\left( Nd+Md \right)+O(Nkd)$. The GCN computation grows linearly with the number of layers $L$, the neighborhood matching computation depends on the number of neighbors $k$, and the cost of the alignment computation is determined by the number of entity pairs $M$. The GCN computation is also a good example of GCN computation. It is worth mentioning that the computational cost may rise significantly in the case of dense graphs ($k\approx N$) or large-scale entity comparisons ($M\approx N^{2}$).

**Comment 3:** *Given the model's complexity, it is unclear how it scales to larger datasets beyond DBP15K. The authors should provide details on training time, memory usage, and computational requirements.*

**Response: Done.** Following this suggestion, we have performed a detailed derivation of the computational complexity of the model in Section 4.5, illustrating how the overall computational complexity varies with data size, number of network layers, and embedding dimensionality based on each module. Although the model structure is more complex, in theory, its complexity is the same as that of the existing GCN-based methods. Moreover, to further verify the model’s scalability, we have conducted experiments on the relatively sparse dataset SRPRS in Section 5.1. The experimental results show that despite the challenges posed by the sparse data, our model still maintains a good performance, which proves that the model has good scalability and adaptability. In addition, we have recorded the model’s training time and compared it with other methods in the last paragraph of Section 5.3.1. The results show that while our method’s training time is slightly longer, this increment is exchanged for higher alignment accuracy and robustness, thus validating the model’s acceptability for real-world applications.

**Comment 4:** *While the DBP15K dataset is widely used in entity alignment research, it does not fully represent the diversity of real-world knowledge graphs. The authors should evaluate ARNM-DAE2A on additional datasets, such as YAGO, Wikidata, or Freebase, to demonstrate broader applicability.*

**Response: Done.** Thanks for the reviewer’s constructive comment. Following this comment, we have supplemented a new dataset SRPRS in Section 5.1. Specially, since the DBP15K dataset is too dense and the degree distribution is very different from the real-world data, we also chose two cross-linguistic subsets from the relatively sparse SRPRS dataset [33], and comprehensive dataset details are presented in Table 2.

**Supplemented references:**

[33] Guo L, Sun Z, Hu W. Learning to Exploit Long-term Relational Dependencies in Knowledge Graphs. In: Chaudhuri K, Salakhutdinov R, editors. Proceedings of the 36th International Conference on Machine Learning. vol. 97; 2019. p. 2505-2514.

**Comment 5:** *The manuscript lacks a discussion on the potential biases in DBP15K and whether they might influence the reported results.*

**Response: Done.** Thanks for the reviewer’s constructive comment. Following this comment, we have supplemented the robustness with textual noise in the 2^nd^ paragraph of Section 5.3.4. Specially, referring to [39], we test the robustness of the model by artificially introducing different degrees of textual noise, where textual noise includes entity errors, missing words, and additional characters. As shown in Fig. 8, the alignment accuracy of each dataset tends to decrease as the proportion of noise increases, but the magnitude of the decrease is significantly different. In the DBP15K subsets, Hits@1 decreases more gently, which indicates that the model can rely on sufficient structural and attribute information to mitigate the negative impact of noise in the case of richer information. In the SRPRS subsets, the decrease in Hits@1 is more pronounced due to the sparser graphs and less redundant information. This suggests that textual perturbations interfere more with entity alignment in noisy scenarios with sparse data. Overall, the experimental results illustrate that the proposed ARNM-DAE2A is robust under low to moderate noise levels, but there is still room for improvement under high noise and sparse data conditions, and further optimization of the noise processing and information fusion strategies is needed.

**Supplemented references:**

[39] Yoon S, Ko S, Kim T, Kang S, Yeo J, Lee D. Unsupervised Robust Cross-Lingual Entity Alignment via Neighbor Triple Matching with Entity and Relation Texts; 2025. Available from: https://arxiv.org/abs/2407.15588.

**Comment 6:** *The performance of deep learning models is often sensitive to hyperparameter choices. While the manuscript provides a brief discussion on hyperparameter tuning (e.g., entity embedding dimensions, GCN layers, attention heads), it does not analyze how robust the model is to hyperparameter variations.*

**Response: Done.** Thanks for the reviewer’s constructive comment. Following this comment, we have supplemented the robustness with different hyperparameters in the 1^st^ paragraph of Section 5.3.4. Specially, as shown in Fig. 7(a), embedding dimension in the range of 200 to 700 , the five subsets show a gradual upward trend and stabilize at 500 to 600 dimensions. This suggests that as the embedding dimension increases, the model can learn richer entity features, thus improving the alignment accuracy. However, when the dimension exceeds a certain threshold, the performance gain gradually diminishes, indicating that the model is more robust to changes in the embedding dimension in the range of 500-600, and the performance degradation is limited even if the parameters slightly deviate from the optimal values. As shown in Fig. 7(b), with respect to the number of GCN layer, the result shows that as the number of GCN layers is increased from 1 to 4, the Hits@1 of the model is significantly improved in both datasets, which suggests that the increase in the number of layers can help to capture deeper graph structure information. However, the model performance slightly decreases when the number of layers exceeds 4, probably due to the effect of the over-smoothing phenomenon, which makes the node representations indistinguishable. This result shows that the model is more sensitive to the number of GCN layers, but within a reasonable range, the appropriate number of layers can effectively improve the model performance, while too many layers may lead to excessive aggregation of information, which may affect the alignment accuracy. As shown in Fig. 7(c), the result shows that in the range of 1 to 8, all the five subsets under perform best in the interval of 3 to 5 heads, followed by a slight decrease in performance as the number of dual attention heads increases. This suggests that an appropriate amount of dual attention heads can help the model capture multi-angle information more comprehensively and enhance entity matching. However, too few attention heads can limit the model’s learning ability, while too many heads may introduce additional noise or lead to a waste of computational resources. Therefore, although the model is robust to parameter variations in the range of 3 to 5 heads, it still needs to be tuned within a reasonable interval to ensure optimal performance.

**Comment 7:** *Some sections, particularly in the experimental results, could benefit from more structured discussions rather than listing numerical results.*

**Response: Done.** Thanks for the reviewer’s constructive comment. Following this comment, we have supplemented more structured discussions in Section 5.3.1 and Section 5.3.2. Due to the length, we will not include detailed supplements in the response letter. Please see Section 5.3.1 and Section 5.3.2 for specific supplements.

**Response to Reviewer 7’s Comments**

**Comment 1:** *It would be helpful if the authors could further clarify the significance of their work on the impact of future applications. The manuscript would be strengthened if the authors could highlight the limitations of the advances from this proposed model and provide further details on the novelty of these new approaches in context of the current landscape.*

**Response: Done.** Following this suggestion, we have supplemented the future work in Section 7. Specially, we plan to explore more proactive fusion mechanisms, such as cross-modal attention or dynamic feature selection-based methods, to enable attribute embedding to adaptively adjust the weights between different entity pairs and improve the robustness of alignment. At the same time, we consider introducing dyadic attention to other association tasks, such as cross-language information retrieval or knowledge graph complementation, to expand its application areas.
